# Profibrotic pathways and atrial cardiomyopathy in persistent atrial fibrillation

**DOI:** 10.6061/clinics/2016(10)12

**Published:** 2016-10

**Authors:** Uğur Canpolat

**Affiliations:** Hacettepe University Faculty of Medicine, Department of Cardiology, Ankara, Turkey

I enjoyed reading the interesting article by Wu et al. 1, in which the authors demonstrated a significant relationship between matrix metalloproteinase (MMP)-9, a profibrotic and proinflammatory molecule, and recurrence of persistent atrial fibrillation (AF) after catheter ablation. The authors also reported that left atrial (LA) diameter and a history of AF were independent predictors of AF recurrence after ablation therapy.

In recent years, the term fibrotic atriocardiomyopathy (FACM) has been proposed to define the atrial remodeling process resulting in atrial myocyte abnormalities and causing fibrosis in patients with lone AF 2. Previous studies have confirmed that the majority of patients with AF without apparent structural heart disease (referred to as ‘lone' AF) exhibit a chronically fibrotic bi-atrial substrate 3. Electroanatomical mapping and cardiac magnetic resonance imaging with delayed enhancement (DE-MRI) are clinical tools that are potentially useful for diagnosing FACM 4,5. Atrial fibrosis is a critical pathophysiological abnormality of the atrial substrate; is associated with electrical, contractile, and structural remodeling of atrial tissue; and is involved in both the initiation and the maintenance of AF 6,7. The MMP family regulates the degradation of atrial collagen and other extracellular matrix molecules. Impairments in this well-organized pathway occur during atrial remodeling and fibrosis 8. As a member of the MMP family, MMP-9 is known to be a marker of enhanced proinflammatory and profibrotic pathway activities. The study by Wu et al. 1 included only patients with persistent AF without apparent structural heart disease and demonstrated an association between MMP-9 levels and AF recurrence after catheter ablation for persistent AF; however, their study lacked direct evidence regarding the mechanisms underlying AF recurrence. The authors should have commented on their procedural electroanatomical mapping data, which may have highlighted the mechanism underlying AF recurrence, in the event that the MMP-9 levels correlated with the area of LA scar tissue (indicating LA fibrosis). Furthermore, their study lacked data on whether the type of persistent AF was primary or secondary in these patients. It was recently proposed that persistent AF can occur either as a sustained arrhythmia progressing from paroxysmal AF or as primary persistent AF without any history of spontaneously terminating episodes 9. Researchers have reported that the AF recurrence rate after catheter ablation was significantly higher in patients with primary persistent AF than in patients with secondary AF progressing from paroxysmal AF. The authors also commented on revolutionary studies demonstrating the failure of using additional lines during catheter ablation to treat persistent AF 10.

In conclusion, serum MMP-9 levels may significantly predict the success rate of catheter ablation therapy in patients with persistent AF, probably by indicating the extent of LA fibrosis. However, additional non-invasive or invasive measures should be used in conjunction with this biomarker to predict the success rate of catheter ablation for the treatment of persistent AF.
